# Comparative Analysis of Cutting Forces, Torques, and Vibration in Drilling of Bovine, Porcine, and Artificial Femur Bone with Considerations for Robot Effector Stiffness

**DOI:** 10.1155/2020/8817422

**Published:** 2020-10-19

**Authors:** Oluseyi Adewale Orelaja, Xingsong Wang, Donghua Shen, Dauda Sh. Ibrahim, Tianzheng Zhao, Umer Sharif, Ishola A. Afiz

**Affiliations:** ^1^School of Mechanical Engineering, Southeast University, Nanjing, China; ^2^Department of Mechanical Engineering, Moshood Abiola Polytechnic, Abeokuta, Ogun, Nigeria; ^3^Department of Mechanical Engineering, Federal University of Agriculture, Abeokuta, Ogun, Nigeria

## Abstract

Bone drilling is known as one of the most sensitive milling processes in biomedical engineering field. Fracture behavior of this cortical bone during drilling has attracted the attention of many researchers; however, there are still impending concerns such as necrosis, tool breakage, and microcracks due to high cutting forces, torques, and high vibration while drilling. This paper presents a comparative analysis of the cutting forces, torques, and vibration resulted on different bone samples (bovine, porcine, and artificial femur) using a 6dof Robot arm effector with considerations of its stiffness effects. Experiments were conducted on two spindle speeds of 1000 and 1500 rpm with a drill bit diameter of 2.5 mm and 6 mm depth of cut. The results obtained from the specimens were processed and analyzed using MATLAB R2015b and Visio 2000 software; these results were then compared with a prior test using manual and conventional drilling methods. The results obtained show that there is a significant drop in the average values of maximum drilling force for all the bone specimens when the spindle speed changes from 1000 rev/min to 1500 rev/min, with a drop from (20.07 to 12.34 N), approximately 23.85% for bovine, (11.25 to 8.14 N) with 16.03% for porcine, and (5.62 to 3.86 N) with 33.99% for artificial femur. The maximum average values of torque also decrease from 41.2 to 24.2 N·mm (bovine), 37.0 to 21.6 N·mm (porcine), and 13.6 to 6.7 N·mm (artificial femur), respectively. At an increase in the spindle speed, the vibration amplitude on all the bone samples also increases considerably. The variation in drilling force, torque, and vibration in our result also confirm that the stiffness of the robot effector joint has negative effect on the bone precision during drilling process.

## 1. Introduction

Bone drilling is a repair technique which involves creating a pilot hole for proper insertion or screwing on the already inserted plate, or for attaching prosthetic devices to provide rigidity and prevent misalignment of the fractured bone [1–3]. The bone is a hard, anisotropic, heterogeneous, and viscoelastic connective tissue that constitute the skeletal system, exhibiting piezoelectric properties due to the complexity of the binding structure in the dry state. However, bone is a poor conductor of heat, with the thermal conductivity of fresh cortical bone at approximately 0.38–2.3 J/m·sK^1^. It means that bone could not dissipate the heat generated immediately when cutting forces are applied on it, and consequently, temperature in the drilled site is increased [4]. According to Currey [5], bone has high stiffness features, therefore tends to break or fracture when subjected to high external forces. Yearly, around seven million car accidents happen in the United State of America, resulting in femur shaft fractures [6], which could be attributed to reckless driving and over speeding on highways. According to Gupta and Tse [7], femur is the longest and strongest bone in the human body, with its fractures occurring in middle-age patients which are due to high energy impact force, while mostly due to low energy or impact fall in aged women.

Bone drilling is a repair process peculiar to a femur fracture, which involves creating a pilot hole for proper insertion or screwing on the already inserted plate to avoid misalignment. However, drilling an accurate hole with minimal cutting force and vibration to prevent cracks, and tool breakage, or with no complication, are essential. Often, surgical drill is operated manually by a surgeon, and in some cases, the operation requires considerable skill and a high degree of mental and utmost concentration. Although the use of robots in the theatre room is so enormous now due to its flexibility, the adequacy and usefulness in various areas of surgical fields are paramount [8]. In this work, Hans Robot model HREF 01-LD010-1000-SI was employed for the drilling. The drilling and navigation were done by the robot effector which complemented the aspects of computer-assisted orthopedic surgery [9] due to high precision level. Improper bone drilling procedure can negate the clinical result due to the following: (1) high cutting force and (2) torques and (3) excessive vibration and (4) type of the cutting tools [10]. High forces, torques, and extreme vibration are caused by improper tooling and use of manual drilling method, resulting to serious complications [11]. This research is based on the critical criteria that affect surgical bone drilling [9, 12–14], as well as checking the relationship between the amount of forces, torques, and vibration while drilling different bone samples with utmost consideration of the robot effector stiffness and possible error limits compensation [15]. In this experiment, bovine bone was used to replace human femur because of the similarity in their properties as described by Poumarat et al. [15, 16]. The determination of the successful surgical drilling method depends mostly on parameters such as force, torque, and vibration [17, 18]. Many researchers have compared surgical drilling of bovine and artificial femur [19], but no one has considered bovine, porcine, and artificial femur with utmost consideration for the stiffness on the effector of the robot arm during their investigations. However, due to low drilling accuracy resulting from weak stiffness and low kinematic accuracy, industrial robot is rarely applied in precision machining process [20]. Therefore, in order to reduce deformation or backlash due to vibration during bone drilling, robot stiffness influence should be considered; hence, this study is focused on measuring and comparing the cutting forces, torque and vibration on the bone while drilling, with consideration of the robot stiffness.

## 2. Methods

### 2.1. The Bone Drilling Set-Up System

**Figure 1:**
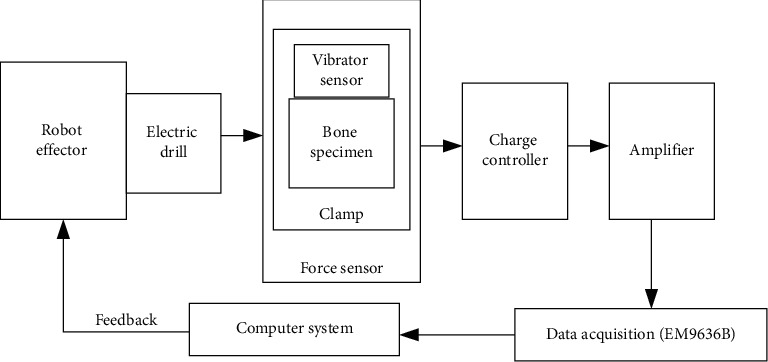
The block diagram of the bone drilling process.

The set-up of the experiment involved 6dof Hans Robot which is a PC-based and single phase 220 V (50–60 Hz, power 1.5 kW) electric motor with a speed control range of 1500 rpm. Also, a four-component dynamometer (Kistler Type 9272 A) was calibrated and mounted on the work platform. The ICAM amplifier was set with the PC software to measure this range of coulomb: ±100 pC and ±10 pC; data acquisition system measured the electrical current signals from the force sensor in accordance to the exerted force and torque across all the axes, a vibrator sensor (accelerometer) of frequency range of 20–30 Hz was also attached to each of the bone specimen to measure the resulted vibration, and a charge controller regulates voltage and a computer system interprets the visible signals. The hardware block diagram is shown in Figure 1.

### 2.2. Bone Samples for Drilling

**Figure 2:**
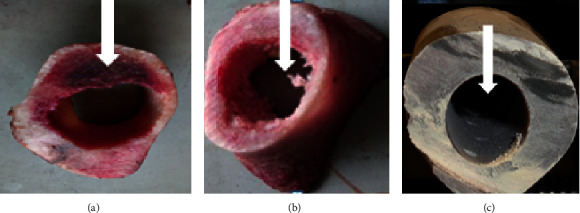
Cross-sectional views of (a) bovine, (b) porcine, and (c) artificial femoral samples.

The bone samples used for the experiments were excised from the middle portion of the bovine and porcine femur (see Figures 2(a) and 2(b)) and obtained from butchers shop (Table 1). The residue tissues on the femur were stripped off to ensure that no defect of any sort is seen on the bones, followed by refrigeration to a temperature of about −20°C before drilling and allowed to thaw at 24 °C ambient room temperature for at least 90 minutes before the drilling. The composite femur (large left femur, Model 3310, Sawbones, Pacific Research Labs, Vashon Island, WA, USA) samples were purchased for the experiments and used as received, as shown in Figure 2(c).

### 2.3. Mechanical Properties of Human Bone, Bovine Bone, and Pig Bone

Mechanical properties of human bone, bovine bone, and pig bone are given in Table 1.

### 2.4. Drilling Method and Mechanical Modeling

The parameters used for drilling in this experiment are provided in Tables 2 and 3. The drill bit diameter, range of drill speed, and feed rates used in this study are widely reported in the literature related to robotic and navigation procedures and applications of bone drilling as suggested by previous works [17, 23–25]. During the drilling, the force, torque, and vibration measurements were recorded in *z*-axis only.

**Figure 3:**
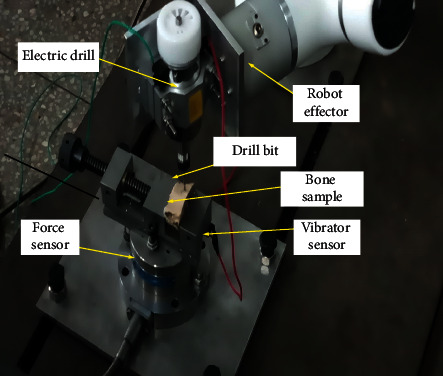
The bone drilling procedure using the 6dof Hans Robot.

The robot-effector's arm was set to drill through the depth of 6 mm for all the specimens, while the drilling force, torque, and bone vibration were measured in *z*-axis only at varying cutting speed of 1000 rpm and 1500 rpm. This procedure was repeated twice to ensure repeatability and error-free.  Figure 3 shows the drilling procedure using the 6dof Hans Robot for bone drilling.

### 2.5. Modeling of Robot Effector Stiffness

**Figure 4:**
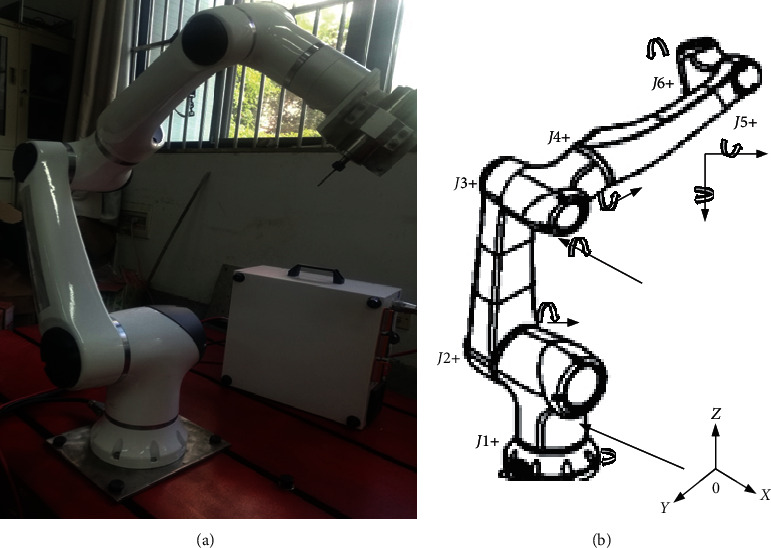
Hans Robot model HREF 01-LD010-1000-SI.

The stiffness of a robot is of great importance to accurately manipulate drilling operation. It shows the accuracy and the rigidity needed by the force effector to drill with less vibration and deflections [26, 27].  Figure 4(a) shows he complete set-up of the Hans Robot manipulation process; however, it is essential to model the stiffness of the Hans Robot when drilling to compensate for errors that could occur due to external forces on the effector. Modeling the stiffness end of the effector is done by applying Jacobian matrix principle to identify the relationship between joint rotation and end effector motion, as shown in Figure 4(b).

#### 2.5.1. Jacobian Matrix

This matrix *J*(*q*) of robot is used to determine the relationship between the joint rotation Δ*q* and end effector motion Δ*X* [28]. The relationship of the robot actuator motion and force exerted on the specimen to be drilled can be obtained as follows:

(1) $$\begin{array}{c} \Delta X=J\left(q\right)\Delta q, \end{array}$$

where *J*(*q*) can also be expressed as representing 6 × 1 (external forces vector) on the manipulator end-point.

(2) $$\begin{array}{c} J_{i,j}\left(q\right)\frac{\partial X\;i\;\left(q\right)}{\partial q_{j}},\;\left(i,j=1,2,\ldots ,6\right), \end{array}$$

and the relationship between joint torques and counter actuator forces/torques to stabilize the external force is illustrated as

(3) $$\begin{array}{c} T=J^{T}\left(q\right)F, \end{array}$$

where *τ*=(*τ*_1_, *τ*_2_, *τ*_3_, *τ*_4_, *τ*_5_, *τ*_6_) represent the 6 × 1 vector of the torques needed to balance the external force during bone drilling. *F*=(*F*_*x* , _*F*_*y* _, *F*_*z*_, *τ*_*yz*_, *τ*_*xz*_, *τ*_*xy*_).

#### 2.5.2. Stiffness Model

The end deflection Δ*X* on the effector caused by external force can be calculated by [29]:

(4) $$\begin{array}{c} F=K_{m}\Delta X, \end{array}$$

where the angular rotation of the joint is given as

(5) $$\begin{array}{c} \tau =K_{m}\Delta X, \end{array}$$

where *K*_*m*_ is the Cartesians stiffness of the effector manipulator and *K*_*θ* _ represents the joint stiffness. However, the partial differentiation of equation (3) with respect to *q* results is

(6) $$\begin{array}{c} \frac{\delta \tau }{\delta q}=\frac{\delta J^{T}}{\delta q}F+J^{T}\frac{\delta F}{\delta X\;\left(q\right)}\frac{\delta X\;\left(q\right)}{\delta q\;}. \end{array}$$

By replacing equation (5), the equation of the joint stiffness can then be summarily written as

(7) $$\begin{array}{c} K_{\theta \;}=K_{c\;}+J^{T}K_{m}J. \end{array}$$

It must be noted that *K*_*c* _=*δJ*^*T*^/*δqF* is the complimentary stiffness of the robot effector due to loading or cutting force as a result of drilling, as stated by Claire Dumas [29], which can then be rewritten as

(8) $$\begin{array}{c} K_{m\;}=J^{-T}K_{m}J^{-1}. \end{array}$$

This summarily equals to

(9) $$\begin{array}{c} \left[\begin{array}{ccc} K_{m11\;\;\;} & \cdots & K_{m16\;\;\;}\\ \vdots & \ddots & \vdots \\ K_{m61} & \cdots & K_{m61} \end{array}\right], \end{array}$$

where *K*_*m* _ is the joint stiffness matrix.

Δ*θ* is the model for dynamic manipulator effector stiffness of the robot effector. By further analysis, the stiffness of the effector manipulator can also be remodeled as [30]

(10) $$\begin{array}{c} K_{c\;}=\;\left[{\frac{\delta J}{\delta \theta_{1}}}^{T}F\;{\frac{\delta J}{\delta \theta_{2}}}^{T}F\;{\frac{\delta J}{\delta \theta_{3}}}^{T}F\;{\frac{\delta J}{\delta \theta_{4}}}^{T}F\;{\frac{\delta J}{\delta \theta_{5}}}^{T}F\;{\frac{\delta J}{\delta \theta_{6}}}^{T}F\right]. \end{array}$$

Assuming  (*δJ*^*T*^/*δθ*_1_*F*) is a 6×1  column vector, then drilling force on the robot effector is *F*=[*F*_*x*, _*F*_*y*_, *F*_*z*_]^*T*^ and that resulted in deflection during drilling is Δ*X*=[*δx*,  *δy*, *δz*]^*T*^, by substituting equation (6) into equation (3), we obtained

(11) $$\begin{array}{c} \Delta X=K_{X}^{-1}F=g\left(\theta ,K_{\theta i},F\right). \end{array}$$

**Figure 5:**
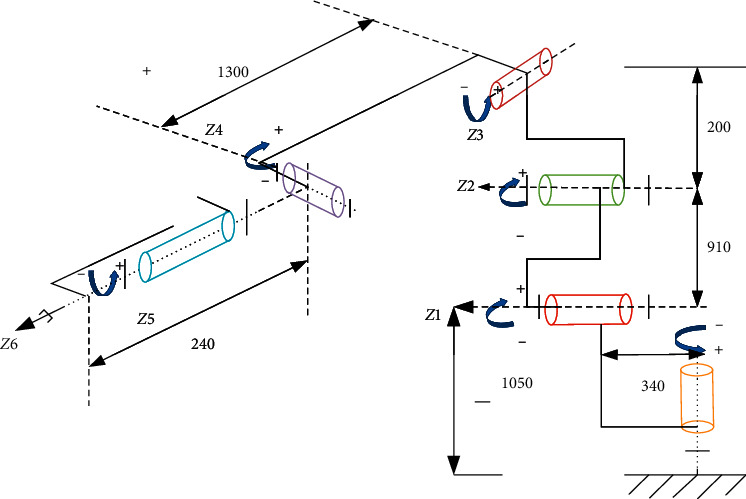
Flow chart of drilling trajectory and stiffness evaluation simulation system.

**Figure 6:**
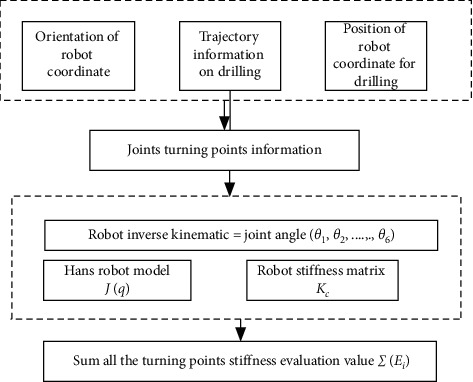
Kinematic chains representation of Hans robot HREF 01-LD010-1000-SI.

The relationship above represents a little deflection of the effector during bone specimen drilling as shown in the experimental set-up in Figure 5. Therefore, joint stiffness *K*_*θ* _ can be written as combinations of stiffness of servo motor  *K*_*d*_, gear shaft  *K*_*j* _, and harmonic reducer *K*_*c* _ of the effector.  Figure 6 shows the schematic diagram of the kinematic chains representation of the Hans Robot used for this experiment.

(12) $$\begin{array}{c} \frac{1}{K_{\theta \;}}=\frac{1}{K_{d\;}}+\frac{1}{K_{j\;}}+\frac{1}{K}. \end{array}$$

With reference to *K*_*θ* _, the stiffness matrix varies as drilling changes, this can be mathematically summarized as

(13) $$\begin{array}{c} \sum^{}=\sum_{i=1}^{6}k_{ii}+\sum_{i=1,j=1,}^{6}i\neq j/k_{ij}/. \end{array}$$

The inertia cutting force in the direction of acceleration causes unbalance force in the robot system which affects the stiffness and possibly given errors or affects its precision.  Table 4 illustrates the robot joint type and components for its manipulations.

### 2.6. Analysis of the Drilling Force

**Figure 7:**
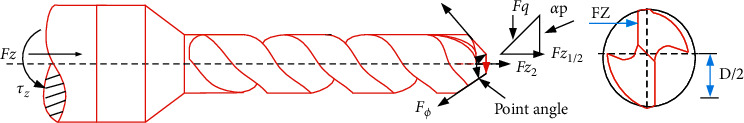
Force on twist drill bit during bone drilling of the bone specimen.

Force analysis of the 6dof Robot arm is presented as a relationship between the effector, torque (*τ*_*z*_) of the electric motor, and the force  (*F*_*z*_) generated. Different forces on a twist drill are shown in Figure 7, where *F*_*z*_ is not fully caused by the *F*_*q*_ components of the cutting force  (*F*_*z*1_) but partially caused by the impacts force on the cutting chisel edge  (*F*_*z*2_). The developed derivatives are dependent on the fact that direct current motor torque is proportional to the motor power, the torque controller of the motor driver has the transfer function *G*(*s*) as

(14) $$\begin{array}{c} G\left(s\right)=\frac{k_{1}}{k_{1}\left(s\right)+1}. \end{array}$$

From Figure 5, *F*_*z*1_ is deduced as

(15) $$\begin{array}{c} \;F_{z1}=2\;F_{q}\cos\alpha_{p}. \end{array}$$

Here, *α*_*p*_ is the inclination angle of force  *F*_*q*_ experienced on the cutting edge of the drill, as shown in Figure 6, which is

(16) $$\begin{array}{c} \alpha_{p}=90^{0}-\frac{2\rho }{2}, \end{array}$$

where 2*ρ* is the point angle of the drill bit.

Torque effect is a factor of the drill diameter and the amount of drilling force *F*_*p*_ that is on the bone specimen type:

(17) $$\begin{array}{c} \tau_{z}=\frac{d}{2}F_{p}. \end{array}$$

When a more substantial size drill is used, then the motor torque will be higher, which will affect the stiffness of the robot effector and the quality of the hole made.

### 2.7. Vibration Analysis

The displacement, *y(t)*, caused by the vibrating bone causes displacement, *y*_*e*_*(t)*, of the vibration meter output attached to the bone as stated by [31] so that the relative displacement *y*_rel_(*t*) is given as follow.:

From the equation of motion,

(18) $$\begin{array}{c} m{\ddot{y}}_{e}\left(t\right)+c\left({\dot{y}}_{e}\left(t\right)-\dot{y}\left(t\right)\right)-k\left({\dot{y}}_{e}\left(t\right)-y\left(t\right)\right), \end{array}$$

where *y(t)* and *y*_*e*_(*t*) are the displacements of the vibrating bone and the mass of the vibrating sensor,

(19) $$\begin{array}{c} m{\ddot{y}}_{\text{rel}}\left(t\right)+c{\dot{y}}_{\text{rel}}\left(t\right)+ky_{\text{rel}}\left(t\right)=-m\ddot{y}\left(t\right), \end{array}$$

(20) $$\begin{array}{c} y_{\text{rel}}\left(t\right)=y_{e}\left(t\right)-y\left(t\right), \end{array}$$

where equation (20) is the relative displacement of the vibration sensor mass with respect to the displacement caused by the vibrating bone during drilling. The solution of equation (20) can then be rewritten as

(21) $$\begin{array}{c} y_{\text{rel}}\left(t\right)=\frac{-m\ddot{y}\left(t\right)/k}{\sqrt{\left({\left(1-r^{2}\right)}^{2}+{\left(2\zeta r\right)}^{2}\right)}}=\frac{r^{2}y\left(t\right)}{\sqrt{\left({\left(1-r^{2}\right)}^{2}+{\left(2\zeta r\right)}^{2}\right)}}. \end{array}$$

Assuming the ratio of the frequency,

(22) $$\begin{array}{c} r\frac{\omega }{\omega_{n}}=\frac{f}{f_{n}}\ll <1\approx 0, \end{array}$$

where *f* is the frequency of the vibrating bone and *f*_*n*_ is the natural frequency of the sensor attached to the bone. Then, the vibration rate of the bone during drilling is given as

(23) $$\begin{array}{c} y_{\text{rel}}\left(t\right)=\;r^{2}y\left(t\right)=\;\frac{\omega^{2}y\left(t\right)}{\omega_{n}^{2}}. \end{array}$$

### 2.8. Data Analysis

**Figure 8:**
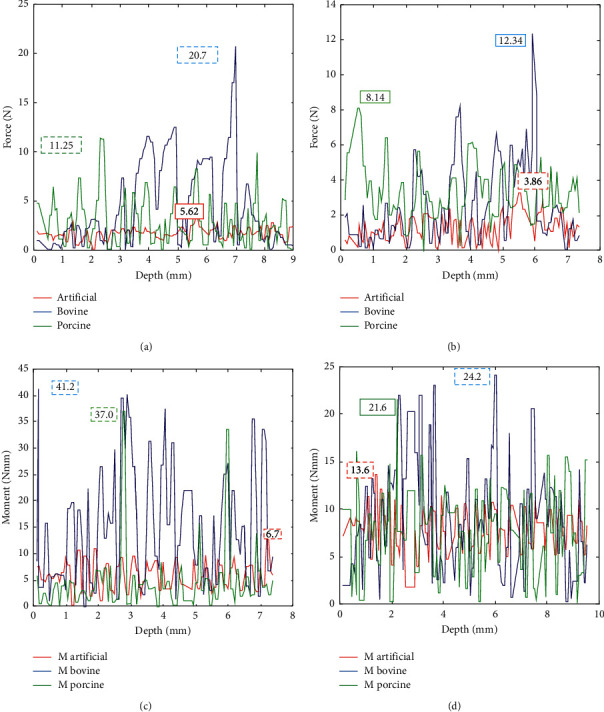
Typical data for drilling at a spindle speed of 1000 rev/min to 1500 rev/min for force and torque for bovine, porcine, and artificial femur, respectively. (a) Force at 1500 rev/min vs. depth. (b) Force at 1500 rev/min vs. depth. (c) Torque at 1000 rpm vs. depth. (d) Torque at 1500 rev/min vs. depth.

**Figure 9:**
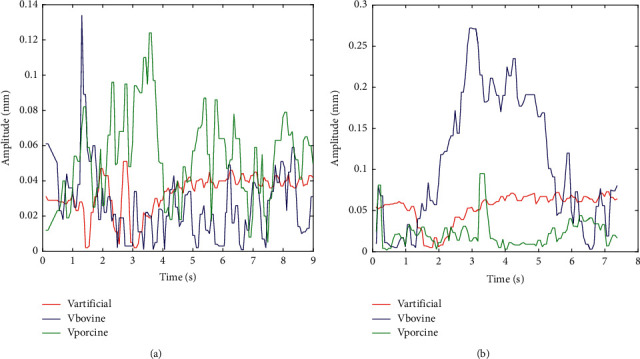
Typical vibration data for drilling at a spindle speed of 1000 rev/min to 1500 rev/min for bovine, porcine, and artificial femur, respectively. (a) Torque at 1000 rpm vs. time. (b) Torque at 1500 rev/min vs. time.

A different set of experiments was conducted to evaluate the drilling force, torque, and vibration resulting on the bone specimens, as shown in Figures 8 and 9, by varying the cutting speed from 1000 rev/min to 1500 rev/min for all the samples with constant feed over specific interval. All the raw data were processed using Microsoft Office Excel 2010 and normalized with MATLAB 2015b to determine the maximum and minimum forces, torque, and vibration during drilling at the two-set spindle speeds [32].  Table 5 also shows the joint and angular range under consideration.

## 3. Results

### 3.1. Maximum Force

At two selected spindle speeds, the result obtained shows that an increase in spindle speed causes a decrease in the average force values on the bovine, artificial femur, and porcine. The following maximum cutting forces of 20.07 N, 5.62 N, and 11.25 N were recorded at 1000 rev/min on each of the specimens as stated: bovine bone, artificial femur, and porcine, respectively. At 1500 rev/min, the maximum cutting force reduced drastically to 12.34 N, 3.86 N, and 8.14 N for bovine bone, artificial femur, and porcine, respectively, as shown in Table 6. This result is amounted to 23.85% (bovine), 22.85% (porcine), and 16% drops (artificial femur), as shown in Figure 8. It was further noted that the bovine bone possessed the highest cutting force which is attributed to its mechanical and material properties.

### 3.2. Maximum Torque

From the torque data obtained, at increase in the spindle speed from 1000 rpm to 1500 rpm, it gives a continuous drop in the maximum torque obtained from 41.2 N·mm to 24.2 N·mm (bovine), from 37.0 N·mm to 21.6 N·mm (porcine), and from 13.6 N·mm to 6.7 N·mm (artificial), as also indicated in Table 6; all these summarily amounted to 25.99% drop (bovine) and 26.27% drop (porcine), except for the artificial femur which has about 33.99% increase, as illustrated in (Figure 8); this increase may be due to the plasticity of the material composition of the artificial femur.

### 3.3. Maximum Vibration

At an increase in the spindle speed from 1000 rev/min to 1500 rev/min, the vibration amplitude increases with time by 33.99% for (bovine), 22.48% for (Porcine), and 30.93% for artificial femur, respectively. This indicated that an increase in the spindle speed affects the stiffness of the robot effector thereby increases the vibration rate due to backlash and less stiffness value, as shown in Figure 9; this extensive increase in the vibration of the artificial femur may also be attributed to the effect of temperature gradient on the material composition due to rise in the drilling speed.

### 3.4. Specimens Quality/Defect Test

A thorough microscopic defect test was conducted on all the bone samples, with no cracks and no necrosis before the test and after, and the drill bit was also checked for excessive or uneven wear.

### 3.5. Robot Stiffness Effector Simulation

**Figure 10:**
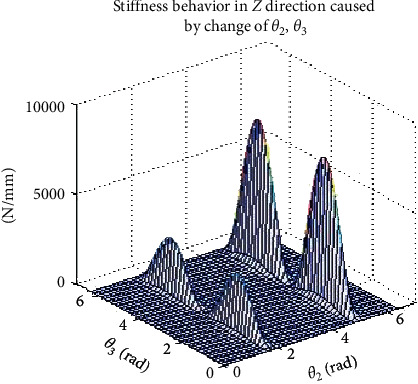
Robot effector stiffness in *Z* direction caused by change from *θ*_2_  to.*θ*_3_.

**Figure 11:**
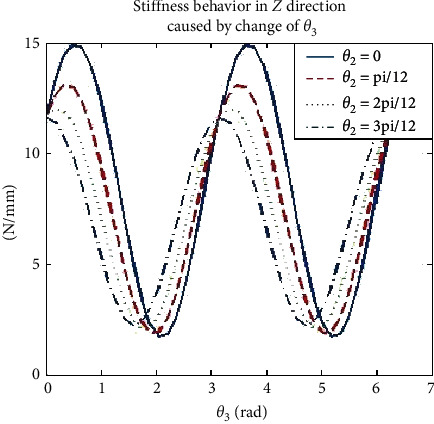
Robot effector stiffness in *Z* direction caused by change of.*θ*_3_.

**Figure 12:**
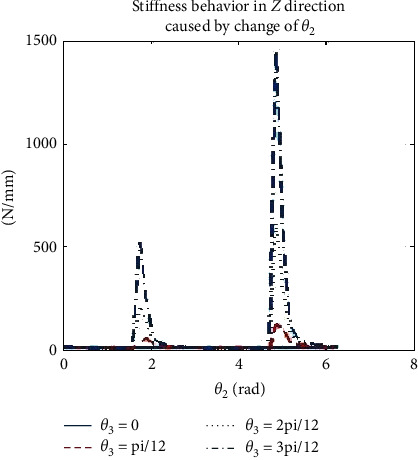
Stiffness behaviors in *Z* direction by change of.*θ*_2_.

Due to the rotational motion of the end effector, there is a change in stiffness which resulted in deformation difference. Hence, it is important to determine the spatial stiffness of the end effector while drilling bone specimen and considering optimization of path drilling plan. From equation (9), it is seen that the stiffness values in *X* and *Y* directions are affected by rotation of joints 1 to 3, while the stiffness value in *Z* direction is also related to joints 2 and 3. Primarily, our attention is based on simulation in the *Z* direction at the effector manipulator joint to stabilize one joint and rotate the other two joints while drilling to observe the effector stiffness. However, Table 5 shows us the range of variations in each joint during the simulation. The spatial behavior of stiffness in the *Z* direction can be evaluated by changing the value of *θ*_2_ and *θ*_3_ at the effector end while drilling. The stiffness simulation at the robot effector is seen in Figure 10, stiffness in *Z* direction also causes an increase of *θ*_3_ which leads to a sinusoidal change, as illustrated in Figure 11, and the amplitude of fluctuation is from 2 “N/mm” to 15 “N/mm. This implies that the phase position and peak value of this sinusoidal change is sensitive to change of *θ*_2_ , as illustrated in Figure 12.

## 4. Discussion

### 4.1. Broad Findings

The experimental investigation and analysis showed that maximum forces were obtained at a spindle speed of 1000 rev/min and substantially dropped when the speed increased to 1500 rev/min. These indicated that the lower the speed, the higher the cutting force and the higher the chance of complications such as tool breakage or microcracks on the bone. On the contrary, an increase in speed reduced the torque as seen on all the samples. From earlier data obtained, the graph in Figure 8 shows that both animal and artificial femurs give a substantial rise in the force on the drill bit entering the bone. This is similar to the force and torque fluctuation response pattern as reported by Lee et al. [13, 35, 37]. For the changes at the spindle speed from 1000 rev/min to 1500 rev/min, there is a significant drop in the average values of maximum drilling force for all the bone specimens with a drop from 20.07 to 12.34 N, approximately 23.85% for bovine, (11.25 to 8.14 N) with 16.03% for Porcine, and (3.86 to 5.62 N) with 33.99% for artificial femur. The maximum average values of torque also decrease from 41.2 to 24.2 N·mm (bovine), 37.0 to 21.6 N·mm (porcine), and 13.6 to 6.7 N·mm (artificial femur), respectively which is close to the result obtained by [13, 36, 38]. At an increase in the spindle speed from 1000 rev/min to 1500 rev/min, it increases the vibration amplitude with time by 33.99% for bovine, 22.48% for porcine, and 30.93% for artificial femur, respectively. This indicated that an increase in the spindle speed affects the stiffness of the robot effector thereby increases the vibration rate, as shown in Figure 9. An increase in the spindle speed from 1000 rev/min to 1500 rev/min increases the rate of depth of cut and reduces the time of cut in all the samples, although the rate of cut also depends on the materials properties of each specimen. The depth of cut in the artificial femur is greatly influenced by increase in speed due to temperature rise on the drill bit which influenced the plasticity behavior of its chemical and materials composition. Generally, the unstable cutting force and torque obtained while drilling can be attributed to the low stiffness of the robot effector; this gives space for a little backlash and vibration during drilling. These results, however, showed a significant variance in the force-torque relationship with the bovine, artificial femur, and porcine and are also used to compare similarities in their parameters and properties which could make them as an experimental substitute to human bone. Force result obtained from the bovine femur is close to the range of findings of Lee et al. [35] which confirms a similarity in the properties of the human bone. From the simulation, the stiffness in *Z* direction can be evaluated by changing the value of *θ*_2_ and *θ*_3_ at the effector end while drilling; also, a little increase of *θ*_3_ could lead to vibration or an unstable effector manipulator during drilling. All the results obtained when compared with prior studies revealed that the robot stiffness has impending effects on the force, torque, and vibration of the bone during drilling.

## 5. Conclusion

A comparative study carried out revealed that both porcine and artificial femur samples have different forces and torques response at a different cutting speed, which are not within the specified range of cutting force and torque applicable for drilling human bone with an exception of bovine bone which has the force range close to the reported finding by [17]. These variances revealed that there is possibly remarkable change in the material properties of both porcine and artificial femur and cannot be a good substitute for human bone unlike bovine for experimental purposes. However, this simulation addressed the effect and behavior of joint rotation on end effector stiffness during bone drilling. The simulation results also show serious mutations during the joint rotation and a sharp peak end curve was generated. Our findings are limited to effector stiffness only, considering all joints will be too complex and out of focus in this study; however, the result evaluated can be regarded as a reference to later research on robot stiffness. To the best of our knowledge, no experiment was done using the 6dof Robot effector to evaluate and compare forces, torques, and vibration in bovine, porcine, and artificial femur drilling, taking into considerations of the stiffness of the effector joint, making this study to be used as a benchmark for further experimental and analytical research on drilling bones for proper orthopedic procedures.

## Figures and Tables

**Table 1:** Prior bone fracture mechanics properties test and values according to [21, 22].

Bone type	Shear strength (Mpa)	*K*_*C*_ (Mpa m^1/2^)	Specific heat (J/kg K)	Energy required	Speed	Test-type
Bovine bone	65–71	3.21	2.58	*G*_JC_ = 1.4–2.6	Slow	SENT
Human bone	82	2.4–5.3	1150	–	Slow	CT
Pig bone	75	–	1330	–	Slow	CT

**Table 2:** Drilling parameters.

Machine speed (rpm)	Drill diameter (mm)	Feed (mm/min)	Depth of cut (mm)	Point angle (°)
1000, 1500	2.5	Set at 110 mm/min	Set at 6 mm for all	118

**Table 3:** Specimen parameters.

Specimens	Bovine	Porcine	Artificial femur
Density	1.193 g/cm^3^	1.013 g/cm^3^	1.86 g/cm^3^
Marrow diameter	32.45 mm	12.05 mm	18 mm
Bone thickness	5.5 mm	5.68 mm	5.91 mm
Specimen length	34 mm	22 mm	35 mm

**Table 4:** Component parts of each joint of 6dof Hans Robot.

Joint	Components
1	Servo motor⟶gear shaft⟶harmonic reducer
2	Servo motor⟶gear shaft⟶harmonic reducer
3	Servo motor⟶gear shaft⟶harmonic reducer
4	Servo motor⟶gear shaft⟶harmonic reducer
5	Servo motor⟶gear shaft⟶conveyor gear shaft⟶harmonic reducer
6	Servo motor⟶gear shaft⟶conveyor gear shaft⟶harmonic reducer

**Table 5:** Variation range of each joint under consideration.

Joint	*θ*_1_	*θ*_2_	*θ*_3_
Range	0 ~ (*π*/2)	0 ~ 2*π*	0 ~ 2*π*

**Table 6:** Comparison of the present experimental results with prior experimental data for surgical drilling into human and animal bone.

Research type	Type specimen	Type result force (N)
Present	Bovine femur	12.34 to 20.07
Present	Porcine femur	8.14 to 11.25
Present	Artificial femur	3.86 to 5.62
Tsai et al. [33].	Human femoral trochanter (cancellous)	1 to 1.5
Tsai et al. [33].	Human femoral trochanter (cortical)	0 to 50
Powers [12]	Porcine vertebra	0.6 to 29.6
Alams et al. [13]	Bovine femoral shaft	25 to 85
Hillery et al. [34]	Bovine tibial shaft	24 to 48
Lee et al. [35]	Bovine tibial shaft	0 to 20
Troy.MacAvelia et al. [17]	Human femoral shaft	140.2 to 186.3
Troy.MacAvelia et al. [17]	Artificial femoral shaft	67.2 to 53.3

Torque (N·mm)		
Present	Bovine femur	24.2 to 41.2
Present	Porcine femur	21.6 to 37.0
Present	Artificial femur	6.7 to 13.6
Tsai et al. [33]	Human femoral trochanter (cancellous)	2 to 120
Tsai et al. [33]	Human femoral trochanter (cortical)	0 to 10
Troy.MacAvelia et al. [17]	Human femoral shaft	16.9 to 16.
Troy.MacAvelia et al. [17]	Artificial femoral shaft	42.9 to 8.4
Alams et al. [13]	Bovine femoral shaft	10–23
Allotta et al. [36]	Porcine femoral shaft	55
Hillery et al. [34]	Bovine tibial shaft	10 to 14.5
Lee et al. [35]	Bovine tibial shaft	0 to 38

## Data Availability

The data used to support the findings of this study are available from the corresponding author upon request.

## References

[1] Orelaja O. A. Vibration reduction, characterization of drill bit and femur bone to forces during robotic-assisted drilling using model soft fixture embedded with pressurized-air damper.

[2] Orelaja O. A., Xingsong W., Kaiwei M., Tianzheng Z., Dauda S. I., Umer S. Experimental investigation of relationship between cutting force, vibration frequency and temperature gradient during robotic assisted bone drilling.

[3] Lee J., Rabin Y., Ozdoganlar O. B. (2011). A new thermal model for bone drilling with applications to orthopaedic surgery. *Medical Engineering & Physics*.

[4] Shakouri E., Mohammad H. S., Mehdi M., Shaghayegh S. (2014). Experimental and analytical investigation of the thermal necrosis in high-speed drilling of bone. *Proc IMechE Part H:Journal of Engineering in Medicine*.

[5] Currey J. D. (1984). Effects of differences in mineralization on the mechanical properties of bone. *Philosophical Transactions of The Royal Society Of London Series B-Biological Sciences*.

[6] BTS (2007). Bureau of transportation statistics annual report. http://www.bts.gov.

[7] Gupta A., Tse K. M. Finite element analysis on vibration modes of femur bone.

[8] Bertelsen A., Melo J., Sánchez E., Borro D. (2013). A review of surgical robots for spinal interventions. *The International Journal of Medical Robotics and Computer Assisted Surgery*.

[9] Jacob R., Mitchell L., Mika S., Blake H., Jacob Rosen B. H., Satava R. M. (2011). Developing a surgical robot from a concept to a transatlantic teleoperation experiment. *Surgical Robotics-Systems Applications and Visions*.

[10] Hirt U., Auer J. A., Perren S. M. (1992). Drill bit failure without implant involvement - an intraoperative complication in orthopaedic surgery. *Injury*.

[11] Sugita N., Mitsuishi M. (2009). Specifications for machining the bovine cortical bone in relation to its microstructure. *Journal of Biomechanics*.

[12] Powers M. J. (2006). The mechanics of bone drilling:experiments and finite predictions.

[13] Alam K., Mitrofanov A. V., Silberschmidt V. V. (2009). Finite element analysis of forces of plane cutting of cortical bone. *Computational Materials Science*.

[14] Karalis T., Galanos P. (1982). Research on the mechanical impedance of human bone by a drilling test. *Journal of Biomechanics*.

[15] Branko K., Miomir V. (1994). Calibration and accuracy of manipulation robot models—an overview. *Mechanism and Machine Theory*.

[16] Poumarat G., Squire P. (1993). Comparison of mechanical properties of human, bovine bone and a new processed bone xenograft. *Biomaterials*.

[17] MacAvelia T., Ghasempoor A., Janabi-Sharifi F. (2012). Force and torque modelling of drilling simulation for orthopaedic surgery. *Computer Methods in Biomechanics and Biomedical Engineering*.

[18] Yu K., Iwata S., Ohnishi K., Usuda S., Nakagawa T., Kawana H. Modeling and experimentation of drilling vibration for implant cutting force presenting system.

[19] MacAvelia T., Salahi M., Olsen M. (2012). Biomechanical measurements of surgical drilling force and torque in human versus artificial femurs. *Journal of Biomechanical Engineering*.

[20] Yonghua C. H., Dong F. H. (2013). Robot machining recent development and future research issues. *International Journal of Advanced Manufacturing Technology*.

[21] Yuehuei H. A. R. (1999). *A Draughn Mechanical Testing of Bone and the Bone-Implant Interface*.

[22] Chen H. L., Gundjian A. A. (1976). Specific heat of bone. *Medical & Biological Engineering*.

[23] O’Toole M. D., Bouazza-Marouf K., Kerr D., Gooroochurn M., Vloeberghs M. (2009). A methodology for design and appraisal of surgical robotic systems. *Robotica*.

[24] Paula G. (2011). Surgical robotics: reviewing the past, analysing the present, imagining the future. *Robotics and Computer-Integrating Manufacturing*.

[25] Lee J., Huh S. J., Lee H. J. (2018). Experimental determination of thermal conductivity of cortical bone by compensating heat loss in parallel plate method. *International Journal of Precision Engineering and Manufacturing*.

[26] Alici G., Daniel R. W. (1994). Static friction effects during hard-on-hard contact tasks and their implications for manipulator design. *The International Journal of Robotics Research*.

[27] Bruyninckx H., De Schutter J. (1996). Specification of force-controlled actions in the task frame formalism-a synthesis. *IEEE Transactions on Robotics and Automation*.

[28] Frankle R. T. (1976). Nutrition education in the medical school curriculum: a proposal for action: a curriculum design. *The American Journal of Clinical Nutrition*.

[29] Alici G., Shirinzadeh B. (2005). Enhanced stiffness modeling, identification and characterization for robot manipulators. *IEEE Transactions on Robotics*.

[30] Frank J., Gritzbach B., Winter C., Maier B., Marzi I. (2010). Computer-assisted femur fracture reduction. *European Journal of Trauma and Emergency Surgery*.

[31] Jyoti K. S. (2015). *Vibration Analysis, Instruments,and Signal Processing*.

[32] Bertollo N., Walsh W., Klika V. (2011). Drilling of bone: practicality, limitations and complications associated with surgical drill-bits. *Biomechanics Application*.

[33] Tsai M.-D., Hsieh M.-S., Tsai C.-H. (2007). Bone drilling haptic interaction for orthopedic surgical simulator. *Computers in Biology and Medicine*.

[34] Hillery M. T., Shuaib I. (1999). Temperature effects in the drilling of human and bovine bone. *Journal of Materials Processing Technology*.

[35] Lee J., Gozen B. A., Arda O., Ozdoganlar O. B. (2012). Modeling and experimentation of bone drilling forces. *Journal of Biomechanics*.

[36] Allotta B., Giacalone G., Rinaldi L. (1997). A hand-held drilling tool for orthopedic surgery. *IEEE/ASME Transactions on Mechatronics*.

[37] Powers M. J. (2006). *The Mechanics of Bone Drilling: Experiments and Finite Predictions*.

[38] Troy MacAvelia M. S., Olsen M., Crookshank M., Emil H. (2012). Schemitsch, ahmad ghasempoor, farrokh janabi-sharifi and rad zdero, biomechanical measurements of surgical drilling force and torque in human versus artificial femurs. *Journal of Biomechanical Engineering*.

